# Hospital costs impact of post ischemic stroke dysphagia: Database analyses of hospital discharges in France and Switzerland

**DOI:** 10.1371/journal.pone.0210313

**Published:** 2019-01-10

**Authors:** Natalia Muehlemann, Baptiste Jouaneton, Lucie de Léotoing, Jean-Jacques Chalé, Jérôme Fernandes, Georg Kägi, Hakan Sarikaya, Marcel Arnold

**Affiliations:** 1 Nestlé Health Science, Epalinges, Switzerland; 2 HEVA; Lyon, France; 3 Finance Department, Geneva University Hospital, Geneva, Switzerland; 4 Groupe OC Santé, Montpellier, France; 5 Department of Neurology, Kantonsspital St. Gallen, St. Gallen, Switzerland; 6 Department of Neurology, University Hospital of Berne, Berne, Switzerland; University of Bologna, ITALY

## Abstract

**Introduction:**

Oropharyngeal dysphagia is frequent in hospitalized post-stroke patients and is associated with increased mortality and comorbidities. The aim of our analysis was to evaluate the impact of dysphagia on Length of Hospital Stay (LOS) and costs. The hospital perspective was used to assess costs.

**Methods:**

Hospital discharge databases comparing hospital stays for ischemic stroke associated with dysphagia vs stroke without dysphagia in France and Switzerland were analyzed. The French Medical Information System Program (PMSI) database analysis focused on 62’297 stays for stroke in the public sector. 6’037 hospital stays for stroke were analyzed from the Swiss OFS (Office fédéral de la statistique: Statistique des coûts par cas 2012) database. Diagnosis codes and listing of procedures were used to identify dysphagia in stroke patients.

**Results:**

Patients with post-stroke dysphagia accounted for 8.4% of stroke hospital stays in Switzerland, which is consistent with recently reported prevalence of dysphagia at hospital discharge (Arnold et al, 2016). The French database analysis identified 4.2% stays with post-stroke dysphagia. We hypothesize that the difference between the Swiss and French datasets may be explained by the limitations of an analysis based on diagnosis and procedure coding. Patients with post-stroke dysphagia stayed longer at hospitals (LOS of 23.7 vs. 11.8 days in France and LOS of 14.9 vs. 8.9 days in Switzerland) compared with patients without post-stroke dysphagia. Post-stroke dysphagia was associated with about €3’000 and CHF14’000 cost increase in France and Switzerland respectively.

**Discussion:**

In this study post-stroke dysphagia was associated with increased LOS and higher hospital costs. It is difficult to isolate the impact of dysphagia in patients with multiple symptoms and disabilities impacting rehabilitation and recovery. After adjusting for confounding factors by matching stays according to age, sex and stroke complications, post-stroke dysphagia association with increased LOS and higher hospital costs was found to be independent of sensory or motor complications.

**Conclusion:**

Post-stroke dysphagia is associated with increased length of hospital stay and higher hospital costs.

## Introduction

Post-stroke oropharyngeal dysphagia is prevalent in hospitalized patients and is associated with increased mortality and comorbidities [1–3]. A recent study on a tertiary stroke center with modern stroke care pathways showed that dysphagia still affects a substantial proportion of stroke patients and has a large impact on clinical outcome (more frequent pneumonia, less often discharged home and living at home at 3 months) and resource utilization (longer stay at monitored stroke unit beds, increased use of antibiotics and chest radiographs, more frequent use of nasogastric tube feeding) [4].

A recent US study of post-stroke dysphagia found that one-year attributable cost to Medicare for patients with dysphagia post-ischemic stroke was $4,510 higher than that for patients without dysphagia [5].

Though dysphagia has impact on hospital and post-discharge resource consumption, the initial burden of dysphagia diagnosis and management is undertaken by acute care especially by stroke units. European certification for Stroke Unit calls for systematic screening for dysphagia in hospitalized stroke patients.

The objective of our analysis was to evaluate the impact of dysphagia on Length of Hospital Stay (LOS) and costs from the hospital perspective. We focused on two different healthcare systems with mandatory health insurance and Diagnosis Related Groups (DRG) based system for acute care funding: France and Switzerland.

In France, a DRG-based payment system (called T2A, Tarification à l’activité) was introduced in 2004/2005 with the major objectives of improving hospital efficiency and improving transparency of hospital activity and management.

In Switzerland, although hospital remuneration was based primarily on fee-for-service (FFS) before 2012, a smaller group of hospitals already used a reimbursement system similar to the AP-DRG (All-Patient DRG) as a precursor of the SwissDRG, introduced in 2012 in all acute care hospitals in Switzerland.

## Methods

### Database

Hospital discharge databases comparing hospital stays for stroke associated with dysphagia vs stroke without dysphagia in France and Switzerland database were analyzed:

- French Medical Information System Program (Programme de Médicalisation des Systèmes d’Information—PMSI) 2012 database
- Swiss OFS (Office fédéral de la statistique: Statistique des coûts par cas) 2012 database

The French PMSI-MCO (médecine, chirurgie, obstétrique) is an exhaustive medico-administrative hospital discharge database which covers all overnight or day hospitalizations in the public and private sectors involving short-term stays in medical, surgical or obstetric facilities. It collects information on the patients and their diagnoses (primary diagnoses (DP—diagnostic principal /DR—diagnostic relié) and comorbidities or complications (DAS—Diagnostic associé significatif)) in CIM10 (Classification internationale des maladies 10ème version) and surgical/interventional procedures (CCAM–Classification Commune des Actes Médicaux). Detailed production costs were issued from ENCC (Echelle Nationale des Coûts à méthodologie Commune), a sample of more than 150 private and public hospitals.

The MS (Statistique médicale des hôpitaux) database collects all patients’ stays in Switzerland. The information gathers patient’s characteristics, diagnoses (ICD10-GM - Internationale statistische Klassifikation der Krankheiten und verwandter Gesundheitsprobleme, 10. Revision, German ModificationDie "Internationale statistische Klassifikation der Krankheiten und verwandter Gesundheitsprobleme" (ICD-10) wird von der) which can be HD (Hauptdiagnosen, main diagnosis—the condition which requested the highest amount of resources during the hospital stay) or ND (Nebendiagnosen, secondary diagnosis—comorbidities or complications), medical/surgical/interventional procedures (CHOP—Schweizerische Operationsklassifikation), as well as detailed production costs documented in addition. Detailed production costs were issued from the FK (Statistique des coûts par cas) database, a sample of 69 hospitals in the MS database.

### Selection of study cohort

CIM10 (French adaptation of ICD10) and ICD10-GM (International Classification of Diseases– 10th version–German Modification) diagnosis codes as well as CCAM (Classification Commune des Actes Médicaux, the French listing of all medical and surgical procedures) and CHOP (the Swiss listing of medical/surgical/interventional procedures) were used to identify stroke patients stays with and without dysphagia.

Relevant diagnosis and procedure codes were selected with coding physicians and stroke neurologists.

Hospital stays were selected from the 2012 dataset by combining several ICD10 codes and excluding some diagnoses to identify stroke patients:

- Stays with codes for Cerebral infarction and Sequelae of cerebral infarction (I63 and I69.3) as main diagnoses were included (DP—Diagnostic Principal in France and HD in Switzerland)
- Stays with codes for subarachnoid hemorrhage, intracerebral hemorrhage, other non-traumatic intracerebral hemorrhage, transient cerebral ischemic attacks and related symptoms as secondary diagnoses were excluded (HD in Switzerland and DR (Diagnostic relié) and DAS (Diagnostic Associé Significatif) in France).
- Stays with secondary diagnosis codes other than stroke that could be a cause of dysphagia, such as head and neck cancer, other head and pathologies, Dementia, Parkinson’s disease, Multiple Sclerosis and other Acute Brain Injury, were excluded in order to eliminate other causes of dysphagia.

26’656’833 stays in PMSI 2012 and 1’227’586 stays in MS 2012 were analyzed. Because the French public sector accounted for 97% of all hospitalization for stroke, further analysis of French data focused on the public sector only. 62’297 stays for stroke in the French public sector and 11’091 hospital stays for stroke were identified in France and Switzerland respectively (MS database). Further analysis for Switzerland focused on 6’037 hospital stays for stroke available in the FK database.

To identify hospital stays with dysphagia, we analyzed codes of dysphagia as secondary diagnosis and procedures codes (CCAM in France and CHOP in Switzerland) related to dysphagia, such as PEG (Percutaneous Endoscopic Gastrostomy) or other types of gastrostomy, feeding tube insertion, FEES (Fiberoptic Endoscopic Evaluation of Swallowing) or VFSS (Videofluoroscopic Swallow Study), evaluation and re-education of swallowing using instrumental control and rehabilitation, adaption, compensation or therapy of swallowing.

### Analysis of burden of post-stroke dysphagia

A descriptive analysis of hospital stays for each group (stroke stay with and without dysphagia) was performed based on number of hospital stays and age and sex of patients.

In order to eliminate confounding factors for the comparison, data from both study groups from databases were matched according to age, sex and stroke complications (no complications, motor complication, sensory complications, motor and sensory complications). Stroke stays with motor or sensory complications were identified by secondary diagnosis codes (ICD10 codes).

Comparison of LOS and costs were then performed using the Mann-Whitney test, with and without matching, in order to assess the burden of post-stroke dysphagia. The hospital perspective was used to assess costs (costing year 2013).

## Results

Patients with post-stroke dysphagia accounted for 8.4% of stroke hospital stays in Switzerland. French database analysis identified 4.2% stays with post-stroke dysphagia (Tables 1 and 2).

**Table 1:** Characteristics of stroke patients with dysphagia vs. without dysphagia in France.

PMSI	Stroke with Dysphagia	Stroke without Dysphagia
**Number of stays**	2 624 (4.2%)	59 673 (95.8%)
**Men—Women**	47% - 53%	52% - 48%
**Age**		
**< 65**	19%	27%
**65–85**	48%	49%
**>85**	33%	34%

**Table 2:** Characteristics of stroke patients with dysphagia vs. without dysphagia in Switzerland.

FK	Stroke with Dysphagia	Stroke without Dysphagia
**Number of stays**	510 (8.4%)	5 527 (91.8%)
**Men—Women**	51% - 49%	55% - 45%
**Age**		
**< 65**	17%	30%
**65–85**	57%	52%
**>85**	26%	18%

Post-stroke dysphagic patients stayed longer at hospitals (LOS of 23.7 vs. 11.8 days in France and LOS of 14.9 vs. 8.9 days in Switzerland) as compared to post-stroke patients without dysphagia.

Post-stroke dysphagia was associated with €2’926 and CHF13’959 cost increase in France and Switzerland respectively (Tables 3 and 4).

**Table 3:** Hospital LOS and Costs of Hospital stays for stroke with vs. without dysphagia in France.

PMSI	Stroke with Dysphagia	Stroke without Dysphagia
**Number of stays**	2’624	59’673
**Average LOS, days (SD)**	23.7 (22.1)	11.8 (10.3)
**Average Cost of Hospital Stay, € (SD)**	€8’770 (5’120)	€5’844 (3’220)

**Table 4:** Hospital LOS and Costs of Hospital stays for stroke with dysphagia vs. without dysphagia in Switzerland.

FK	Stroke with Dysphagia	Stroke without Dysphagia
**Number of stays**	510	5’527
**Average LOS, days (SD)**	14.9 (9.8)	8.9 (8.1)
**Average Cost of Hospital Stay, CHF (SD)**	CHF 27’801 (28’155)	CHF13’842 (11’769)

Additional analysis was performed to test for motor and sensory complications of stroke as confounding factors for hospital LOS and costs. The Mann–Whitney test was used to evaluate the impact of dysphagia on hospital LOS and costs for each of four subgroups: stays with no complications, motor complication, sensory complications and stay with both motor and sensory complications. Results in Tables 5 and 6 below for France and Switzerland respectively show that dysphagia is associated with a statistically significant increase in hospital costs and LOS, independent of presence of motor or sensory complications.

**Table 5:** Hospital LOS and Costs of Hospital stays for stroke with vs. without dysphagia by subgroups in France.

	Hospital LOS			Costs		
Complications	Dysphagia	No Dysphagia	p value	Dysphagia	No Dysphagia	p value
**No complications**	21.3	8.4	<0.0001	8'900	4'284	<0.0001
**Motor**	26.5	11.9	<0.0001	11'194	6'079	<0.0001
**Sensory**	24.8	13.5	<0.0001	9'816	6'685	<0.0001
**Motor & Sensory**	16	9.3	<0.0001	6'495	4'537	<0.0001

**Table 6:** Hospital LOS and Costs of Hospital stays for stroke with vs. without dysphagia by subgroups in Switzerland.

	Hospital LOS			Costs		
Complications	Dysphagia	No Dysphagia	p value	Dysphagia	No Dysphagia	p value
**No complications**	16.3	7.5	<0.0001	31'547	10'402	<0.0001
**Motor**	14.7	9.2	<0.0001	25'282	14'167	<0.0001
**Sensory**	12.9	8.7	<0.0001	21'648	11'784	<0.0001
**Motor & Sensory**	15	9.3	<0.0001	30'626	15'968	<0.0001

## Discussion

The reported frequency of post-stroke dysphagia varies considerably depending on the definition and assessment method used as well as the time of dysphagia evaluation.

In this study, patients with dysphagia accounted for 8.4% of post-stroke hospital stays in Switzerland, which is consistent with recently reported 10% frequency of dysphagia at hospital discharge [4]. A recent study on cost of post-stoke dysphagia in Medicare patients also found 9.9% stroke patients with diagnosis of dysphagia according to ICD-9 coded assigned upon discharge from hospital [5]. The French database analysis identified 4.2% stays with post-stroke dysphagia. The French study on prevalence of stroke and its sequelae reported dysphagia prevalence of 13.3% [6]. We hypothesize that the difference between the Swiss and French datasets analyzed and overall lower prevalence of dysphagia in this study may be explained by the limitations of the methodological approach (analysis based on diagnosis and procedure coding).

In this study post-stroke dysphagia was associated with increased LOS and higher hospital costs. It is difficult to isolate the impact of dysphagia in patients with multiple symptoms and disabilities impacting rehabilitation and recovery. After adjusting for confounding factors by matching stays according to age, sex and stroke complications, post-stroke dysphagia association with increased LOS and higher hospital costs was found to be independent of sensory or motor complications (statistically significant result). There was no control for other variables.

A recent US study on cost of post-stroke dysphagia found significant increases in medical expenses. This study reported 1-year cost to Medicare for patients with dysphagia post ischemic stroke was $4,510 higher than that for post-stroke patients without dysphagia when controlling for age, comorbidities, ethnicity, and proportion of time alive [5].

A limitation of our study methodology using the hospital discharge databases was unavailability of data on stroke severity in the database, as potential confounder. Another limitation of our study was reliance on procedure coding to identify patients with dysphagia which can lead to underestimation of the true frequency of the problem. Because bedside clinical examination is usually most frequently used method to assess dysphagia in clinical practice, not all patients with dysphagia are referred to FEES or VFSS. On the other hand, not all patients referred for FEES or VFSS would have confirmed diagnosis of dysphagia by these instrumental methods. On the other hand, conducting analysis on national level reflects real world clinical practice of dysphagia diagnosis and management as compared to controlled study on selected centers. Because dysphagia may not be systematically captured via coding, available procedures codes selection may have led to identification of more severe dysphagia, reflecting cases requiring medical intervention and leading to increased hospital resources consumption.

## Conclusions

This study indicated that patients with post-stroke dysphagia stayed longer at hospitals (LOS of 23.7 vs. 11.8 days in France and LOS of 14.9 vs. 8.9 days in Switzerland) compared with patients without post-stroke dysphagia. Post-stroke dysphagia was associated with €2’926 and CHF13’959 cost increase in France and Switzerland respectively. The increase in hospital costs and LOS in our study was independent of presence of motor or sensory complications.
